# External stimulation: A potential therapeutic strategy for tendon-bone healing

**DOI:** 10.3389/fbioe.2023.1150290

**Published:** 2023-03-31

**Authors:** Shijie Fu, Yujian Lan, Guoyou Wang, Dingsu Bao, Bo Qin, Qiu Zheng, Huan Liu, Vincent Kam Wai Wong

**Affiliations:** ^1^ Faculty of Chinese Medicine, Macau University of Science and Technology, Taipa, Macao SAR, China; ^2^ Department of Orthopedics, Affiliated Traditional Chinese Medicine Hospital of Southwest Medical University, Luzhou, Sichuan, China; ^3^ Dr. Neher’s Biophysics Laboratory for Innovative Drug Discovery, State Key Laboratory of Quality Research in Chinese Medicine, Macau University of Science and Technology, Taipa, Macao SAR, China

**Keywords:** tendon-bone healing, extracorporeal shock wave, low-intensity pulsed ultrasound, mechanical stress, mechanism of action

## Abstract

Injuries at the tendon-bone interface are very common in the field of sports medicine, and healing at the tendon-bone interface is complex. Injuries to the tendon-bone interface can seriously affect a patient’s quality of life, so it is essential to restore stability and promote healing of the tendon-bone interface. In addition to surgical treatment, the healing of tendons and bones can also be properly combined with extracorporeal stimulation therapy during the recovery process. In this review, we discuss the effects of extracorporeal shock waves (ESWs), low-intensity pulsed ultrasound (LIPUS), and mechanical stress on tendon-bone healing, focusing on the possible mechanisms of action of mechanical stress on tendon-bone healing in terms of transcription factors and biomolecules. The aim is to provide possible therapeutic approaches for subsequent clinical treatment.

## Introduction

**FIGURE 1:**
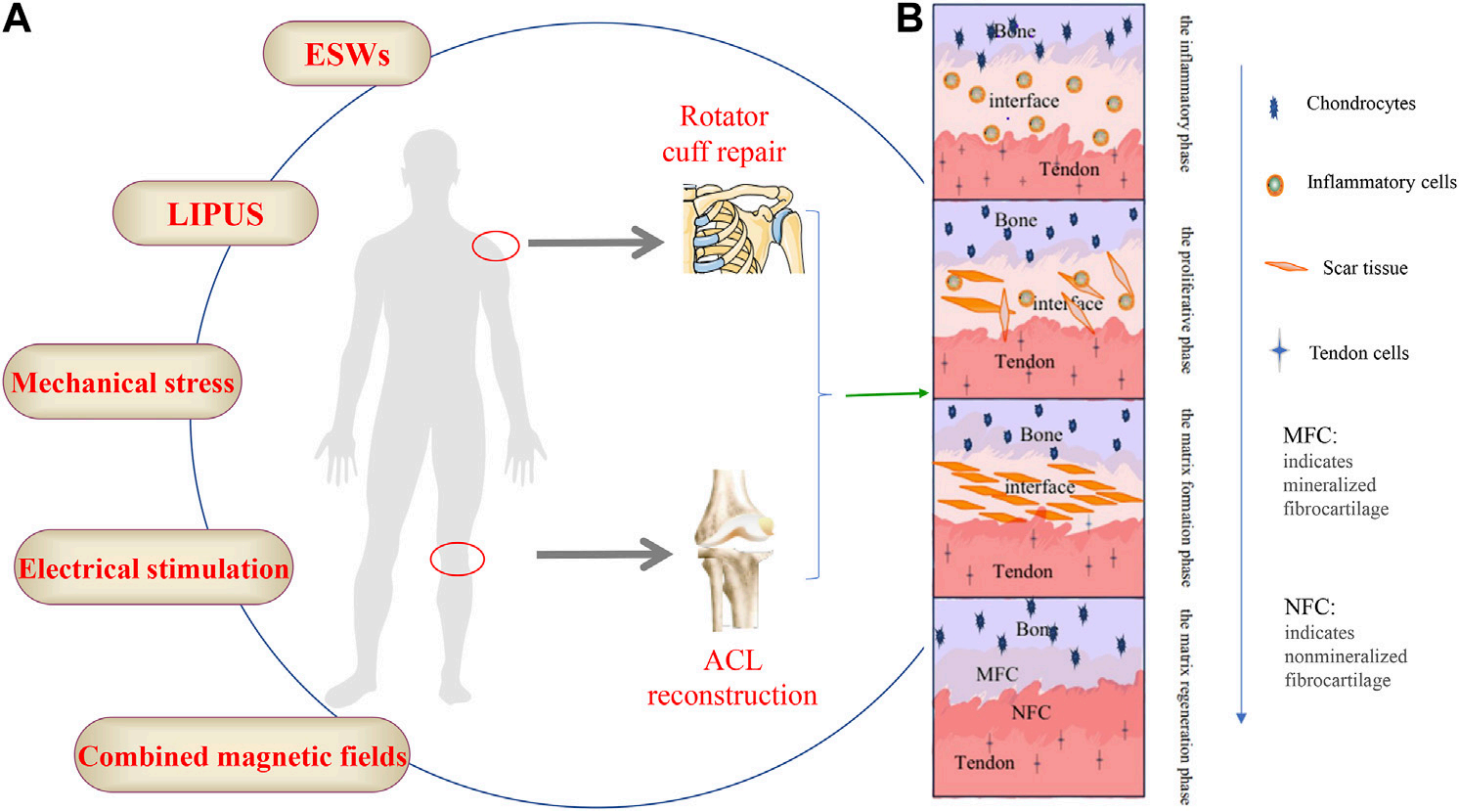
**(A)** The schematic diagram of the external stimulation methods, **(B)** The four stages of the bone-tendon healing process.

Tendon injury is one of the most common clinical diseases, and the global prevalence of tendon disease has been on the rise (Ding et al., 2021). Tendon-bone insertion injuries are also very common, such as rotator cuff and anterior cruciate ligament (ACL) injuries (Xu et al., 2021). And rotator cuff injury and anterior cruciate ligament injury are common soft tissue injuries in the sports medicine field (Tang et al., 2020). Rotator cuff injury is the main cause of shoulder joint pain and weakness, and it even leads to disability (Chalmers et al., 2020; Salvatore et al., 2020). Surgery is the primary treatment for rotator cuff injuries, resulting in increased medical costs, and postoperative retears remain an open problem (Sgroi and Cilenti, 2018; Longo et al., 2020). The preferred treatment for ACL injuries is ACL reconstruction, but follow-up surveys have found that reinjury after reconstruction occurs in far more patients than most other similar treatments (Ahmed et al., 2017; Grassi et al., 2017). Inadequate tendon-bone healing after ACL reconstruction can lead to a variety of secondary symptoms including knee laxity, instability of motion, meniscal and cartilage damage and even early post-traumatic osteoarthritis (Levine et al., 2013; Musahl et al., 2019). Successful tendon-bone healing is the key to restoring physiological function to these injuries, but it remains a clinical challenge (Lebaschi et al., 2018). Tendon adhesions and poor tendon healing not only result in limited movement and pain but also severely affect the patient’s quality of life and can require a second surgery (Chen S. H. et al., 2019; Liu et al., 2019). The normal tendon-bone junction includes four layers of structure: bone, mineralized fibrocartilage, non-mineralized fibrocartilage and tendon (Shi et al., 2020). As early as 1997, researchers outlined the process of tendon-bone healing in a rabbit model, where inflammatory cells could be observed at the tendon bone interface 1 week after surgery, scar tissue appeared after 2 weeks, scar tissue reorganised to form a dense connective tissue matrix after 4 weeks, and by week 6,sharpey’s fibres could be observed (Liu et al., 1997). The process of tendon-bone healing was later divided into four phases: the inflammatory phase, the proliferative phase, the matrix formation phase and the matrix regeneration phase (Lui et al., 2010). This shows that tendon-bone healing is a complex process Figure 1B. Meanwhile, tendon-bone healing is affected by a variety of factors and is prone to scar tissue formation (Lü et al., 2019), the limited mechanical properties of the scar tissue predispose the tendon to re-injury (Yang et al., 2023). In recent years, researchers have been searching for ways to facilitate the tendon-to-bone healing process. Techniques such as biomaterials (Lacheta and Braun, 2021), platelet-rich plasma (PRP) (Peng et al., 2022), cytokines (Zhu et al., 2022), stem cells (Choi et al., 2022) and exosomes (Li et al., 2022; Zou et al., 2023) have been widely used in studies to promote tendon-to-bone healing with promising results. With the development of modern technology and knowledge in biology, ideas and methods for tendon-bone healing have been provided.

The study of external stimulation on tendon-bone healing is being increasingly emphasized, which is of great interest to sports medicine and orthopaedic clinics. Extracorporeal shock waves (ESWs), low-intensity pulsed ultrasound (LIPUS), mechanical loading and other extracorporeal stimulation have all been shown to have varying degrees of therapeutic effects on tendon-bone healing Figure 1A. In recent years, the role of these *in vitro* stimuli on tendon-bone healing has gradually been discovered, especially the potential mechanism of mechanical loading; however, there are still many questions to be investigated. In this review, we focus on the effects of ESWs, LIPUS, and mechanical loading on tendon-bone healing, with an emphasis on the possible mechanisms of action of mechanical loading.

## The role of different types of stimulation on tendon-bone healing

### ESWs

A shock wave is a special, non-linear type of pressure wave with a short rise time (approximately 10 µs) and a frequency ranging from 16 to 20 MHz (Ogden et al., 2001). Currently, extracorporeal shock wave therapy (ESWT) has been shown to have a beneficial effect on tendon healing. For example, ESWT with different parameters in rats with collagenase-induced Achilles tendinitis showed that low-energy ESWT eliminated tendon oedema, swelling and inflammatory cell infiltration, promoted tendon cell proliferation and neovascularization, and promoted transforming growth factor-beta 1 (TGF-β) and insulin-like growth factor-1 (IGF-1) expression during treatment, but high-energy ESWT was not beneficial for tendon repair in rats (Chen et al., 2004) Figure 2A. It can be inferred from this whether ESWT also promotes healing at the tendon bone interface, and it does. In a dose-response study of delayed tendon-bone insertion healing in a rabbit model treated with extracorporeal shockwave, the effect of delayed tendon-bone interface healing was similar in the low-dose ESW and high-dose ESW treatment groups, but did not show evidence of dose dependence, so clinical compliance would have been better (Chow et al., 2012). In another study of partial patellar resection in rabbits to establish a delayed healing model of the patella-patellar tendon complex with a single ESWT treatment, ESWT was found to promote osteogenesis, fibrocartilage regeneration and tissue reconstruction, thereby increasing the effect of tendon-bone repair (Wang et al., 2007). A study using ESWT in rats with osteoarthritis showed that ESWT promotes subchondral bone reconstruction and inhibits cartilage degeneration (Yu et al., 2017). In a study on the improvement of ACL reconstruction by extracorporeal shockwave, tibial tunnel enlargement was significantly reduced in the EWST group and there was reason to believe that ESWT could improve ACL reconstruction through vascular and tissue regeneration (Wang et al., 2014). Additionally, ESWT has an effect on stem cells *in vitro*, giving better potential for the differentiation of stem cells into lipogenic, osteogenic and fibrogenic cartilage (Raabe et al., 2013). Although the biological role of ESWT has been demonstrated in *in vitro* studies, the clinical value of ESWT in human tendinopathy awaits further clinical trials. ESWT has been shown to be effective at a certain stage of tendinopathy and may be indicated in the later stages of degenerative tendinopathy where conservative treatment has failed, but ESWT has no effect on the early stages of tendinopathy (Rees et al., 2009; Zwerver et al., 2011) (Specific information on the effect of ESWT on tendon bone healing is given in Table 1). Few studies have reported on the adverse effects of ESWT on tendon bone healing, and the disadvantages of ESWT in the musculoskeletal system are summarized here, most commonly the appearance of transient skin redness, pain and small hematomas, and in severe cases, possible migraine and fainting (Haake et al., 2002). However, the potential of ESWT needs to be further developed.

**FIGURE 2:**
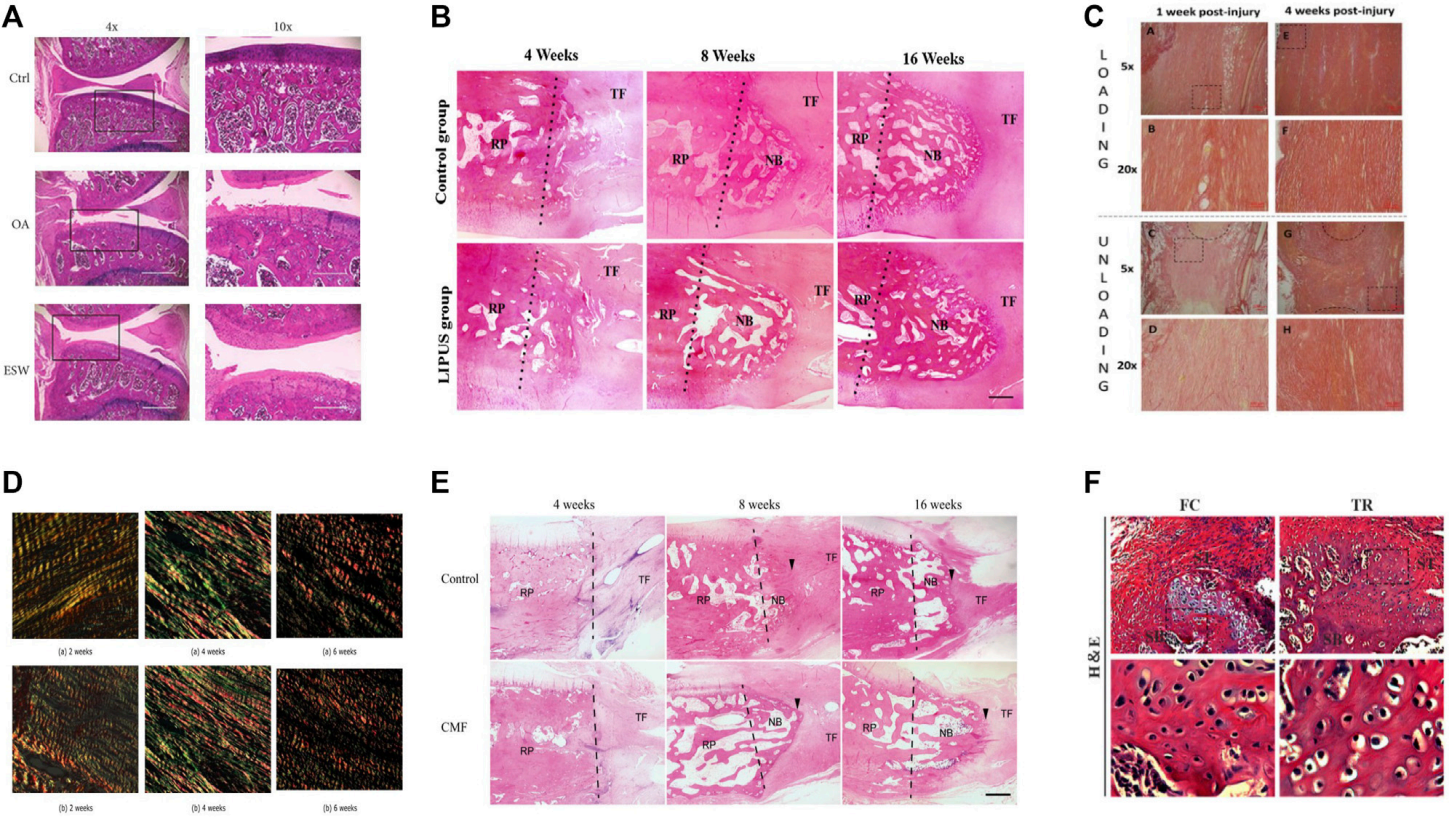
The typical histological or photograph images before and after treatment of each method. **(A)** Before and after ESWT with improved pathology. Reproduced with permission (Yu et al., 2017); **(B)** Comparative histology before and after LIPUS treatment. New bone formation at the proximal patella was observed in both groups at postoperative weeks 8 and 16. The LIPUS group showed more trabecular and marrow cavities than the control group at postoperative week 16. The dotted line represents the surface of osteotomy (NB, newly formed bone; RP, remaining patella; TF, tendon fiber). Reproduced with permission (Lu et al., 2016); **(C)** Before and after comparison chart of mechanical loading treatment. Adipocyte were seen within the callus tissue in both groups at 1 week, but it was more common in the loaded tendons, at 4 weeks, there was less adipocytes in the callus in both groups. Reproduced with permission (Khayyeri et al., 2020); **(D)** Before and after comparison of electrical stimulation treatment. Both collagen 1 and 3 were more highly expressed in the group receiving electrical stimulation than in the control group. Reproduced with permission (Casagrande et al., 2021); **(E)** Before and after comparison chart of combined magnetic field treatment. NB, newly formed bone; RP, residual patella; TF, tendon fiber; the arrowhead, directed at the regenerated fibrocartilage). The CMF group showed more trabecular bone, more marrow cavities, and more advanced remodeling from woven bone to trabecular lamellar bone than the control group. Reproduced with permission (Hu et al., 2015); **(F)** Histological diagram before and after exercise. Mice were allowed for free cage activities after surgery (FC group); Mice received treadmill running initiated on postoperative day 7 (TR group); In comparision to FC group, TR group showed better fibrocartilage regeneration which was characterized by more cartilage-like cells and richer proteoglycans accumulation at repaired site at postoperative week 4 and 8. Reproduced with permission (Liu et al., 2022).

**TABLE 1:** The role of different types of stimulation on tendon-bone healing.

Type	Stimulation parameters	Indications	Effects	Limitations	Reference
ESWs	(0.16 mJ/mm^2^); 0,200,500 and 1,000 impulses	tendinitis	resolved edema, swelling, and inflammatory cell infiltration in injured tendons. Lesion site underwent intensive tenocyte proliferation, neovascularization and progressive tendon tissue regeneration	500 and 1,000 impulses elicited inhibitory effects	Chen et al. (2004)
	0.06 mJ/mm^2^,4 Hz, 1,500 impulses, 0.43 mJ/mm^2^,4 Hz, 1,500 impulses	delayed healing at the patella–patellar tendon complex	Histological assessments confirmed osteogenesis enhancement. Similar effects between the low and high dose for treating delayed tendon–bone insertion healing	Further clinical trials are required as the size of the repair area varies	Chow et al. (2012)
	(0.43 mJ/mm^2^) 4 Hz for 1,500 impulses	delayed healing at the patella–patellar tendon complex	New bone formation better bone mineral status better alignment of collagen fibers; more mature regenerated fibrocartilage zone	imply its application for the above delayed healing conditions characterized with less or no new bone formation, only a single dose was used	Wang et al. (2007)
	1000s, 6 Hz 800s 0.2, 0.4, and 0.6bar	osteoarthritis (OA)	Inhibits cartilage degeneration; promotes the rebuilding of subchondral bone	Low ESW energy levels may have little effect in inducing Wnt5a expression, while excessive ESWT decreased BMMSC vitality	Yu et al. (2017)
	0.298 mJ/mm^2^,1500 impulses	ACL reconstruction	decrease of tibia tunnel enlargement the increases of vascularity and tissue regeneration	the optimal dosage of EWST is unknown	Wang et al. (2014)
LIPUS	1.5 MHz; a 200 ms burst sine wave; 30.0 ± 5.0 mW/cm^2^	the patella–patellar tendon complex	Increased area of new bone; increased bone mineral content	needed to determine the effects of timing and duration of LIPUS treatment	Hu et al. (2014)
	a 200-microsecond burst of 1.5 MHz; 30.0 ± 5.0 mW/cm^2^	the patella–patellar tendon complex	Increased area of new bone; trabecular bone expansion from the remaining patella; regeneration of fibrocartilage layer at the patellar tendon–patella healing junction	A potential dose effect on B-T junction healing was not assessed	Lu et al. (2006)
	1.5 MHz; 1:4 duty cycle and 30 mW/cm^2^	the patella–patellar tendon complex	accelerated the new bone formation and remodeling of new trabecular bone at the bone-tendon junction interface in a rabbit model, and significantly improved the healing quality of tendon–bone insertion injury	LIPUS affects the permeability of the cell membrane; aggravate the inflammation around the normal cells	Lu et al. (2016)
	a 200 us burst of 1.5 MHz; 30.0 ± 5.0 mW/cm^2^	the patella–patellar tendon complex	in early healing phase: regulation of (vascular endothelial growth factor) VEGF expression subsequent: Promoting cartilage formation	More research needed	Lu et al. (2008)
	30 mW/cm^2^; 200 us bursts of sine waves; 1.5 MHz; a 0.2 duty cycle	Rotator cuff injury	Increased bone density at the tendon-bone interface increased VEGF protein expression pattern; Increased osteoblast activity	Dose-dependant effects of LIPUS treatment were not assessed	Lovric et al. (2013)
	1.5 MHz, 1:4 duty cycle, and 30 mW/cm^2^ spatial	Rotator cuff tears	accelerated the accumulation of F4/80 + macrophages at an early stage of tendon–bone insertion healing; promoted the polarization of M2 macrophages	needed to clarify the effect of LIPUS on different macrophage phenotypes	Xu et al. (2022)
Force	A preload of 0.005 N the strain rate of approximately 1 percent/s	the patella–patellar tendon complex	stress shielding significantly decreased the tangent modulus, tensile strength and strain at failure of collagen fascicles	The strain rate used in the present study was relatively low, and the results may be different from those obtained at higher strain rates	Wong et al. (2003)
	A constant force of 2 or 4 N	ACL reconstruction	At week 2 post-operation, the 4 N group still exhibited a better restoration in knee laxity and knee stiffness than the 2 N group; the graft pull-out force and stiffness were also higher	Initial graft tensioning appeared to affect the graft-to-bone interactions, and adverse peri-graft bone changes may negatively affect the strength of graft-to-bone attachment in ACL reconstruction	Fu et al. (2013)
	Preloaded to 0.1N; reconditioned for 10 cycles from 0.1 to 0.5 N; held for 300 s	chronic rotator cuff tears	Repair tension stimulation increases the amount of poor quality scar tissue produced negative effects on tendon-bone healing	this is an animal model study and delayed repair may not be the same as chronic repair in humans	Gimbel et al. (2007)
Electrical stimulation	2 Hz, 1 mA, for 14 days	Achilles tendon tenotomy and tenorrhaphy	collagen 1 and 3 were higher in the group stimulated at 4 and 6 weeks, Low frequency electric stimulation improved healing and increased the quantity of collagen	A comparison of studies on electric stimulation is generally not possible most of the time because of the diversity of methodologies that are employed	Casagrande et al. (2021)
Combined magnetic fields	dynamic sinusoidal magnetic field strength: 400 mG; frequency: 76.6 Hz; magnetostatic field strength: ±200 mG; amphi-magnetic field: ±600 mG	partial patellectomy	The area, length, and bone mineral density of the newly formed bone in the CMF group were significantly greater than the control group at post-operative weeks 8 and 16.The micro-CT results showed that the newly formed bone in the CMF group contained more and thicker trabeculae than the control group at weeks 8 and 16	There are no biomechanical data to measure the healing quality of the BT junction, the size of our samples was not large enough to avoid errors	Hu et al. (2015)
	For alternating current (AC): strength = 400 mG, frequency = 76.6 Hz; for direct current (DC): strength = 6,200 mG, amphimagnetic field = 6,600 mG	partial patellectomy	the newly formed bone and regenerated fibrocartilage zone in the CMF-treated group increased. In the CMF-treated group at postoperative week 16, the amount of proteoglycans was 36.9% more than that of the control group	the relatively small sample size, which may result in statistical errors while arriving at a conclusion	Xu et al. (2014)
Exercise	treadmill running groups maintained a 20 m/min rate run, lane for 60 min per day, 5 days per week	Rotator cuff injury-repair	Early treadmill running increased the expression of NPY at the RC healing site, which might burden the expression of Wnt3a/β-catenin and delay the healing process, inhibition of Y1 receptor with BIBO3304 could promote bone-tendon healing through the Wnt/β-catenin signaling	At the same time, early treadmill running delayed the healing of rotator cuff	Chen et al. (2021c)
	Treadmill speed (5–10 m/min) was increased daily, lane for 20 min per day, 5 days per week	Rotator cuff injury-repair	generation of IL-4, activation of the JAK/STAT signaling pathway in macrophages, the ability of macrophages to polarize towards M2 subtype, and T-B healing quality were significantly enhanced in TR group compared to FC group. Mechanical stimulation could accelerate T-B healing *via* activating the IL4/JAK/STAT signaling pathway that modulates macrophages to polarize towards M2 subtype	the proposed signaling pathway should be validated in human tissue samples	Liu et al. (2022)

### LIPUS

LIPUS is a non-destructive modality in which mechanical energy is transmitted to biological tissues as high frequency acoustic pressure waves through the skin (Ying et al., 2012). LIPUS has been shown to have positive effects on muscles, ligaments, tendons and cartilage (Karnes and Burton, 2002; Takakura et al., 2002; Lai et al., 2021). In a rabbit patellar partial resection model treated with LIPUS, an increase in the area of new bone, bone mineral content, and enhanced repair of the patella-patellar tendon junction were observed (Hu et al., 2014). Interestingly, treatment with LIPUS in the early stages of ACL reconstruction demonstrates that LIPUS may promote tendon-bone healing (Ying et al., 2012). In addition, LIPUS accelerates tendon-bone healing by promoting osteogenesis and tissue reconstruction at the healing junction in a standard patellar partial resection model (Lu et al., 2006). LIPUS treatment was shown to have an anti-inflammatory effect on the healing of the bone-tendon junction and histologically, the LIPUS-treated group showed better remodelling of the lamellar bone and bone marrow cavity (Lu et al., 2016) Figure 2B. LIPUS can also promote scar formation and maturation (Jeremias Junior et al., 2011). However, there are still too few studies on LIPUS in humans. There is no sufficient clinical evidence to evaluate the effect on tendon-bone healing (Ying et al., 2012; Lai et al., 2021). Only some animal studies have shown that LIPUS can promote tendon-bone healing at different stages (Lu et al., 2008; Lovric et al., 2013) (Specific information on the effect of LIPUS on tendon-bone healing is given in the Table 1).

There are also many studies on the mechanisms by which LIPUS promotes tendon bone healing. Several animal studies have noted that LIPUS can improve angiogenesis. LIPUS was used to treat the patella-patellar tendon junction, and histologically significantly higher expression of vascular endothelial growth factor (VEGF) was observed in chondrocytes and osteoblasts than in controls (Lu et al., 2008). LIPUS significantly increased nitric oxide (NO) and hypoxia-inducible factor-1α-mediated expression of VEGF-A in human osteoblasts (Wang et al., 2004). LIPUS can enhance tendon bone healing by stimulating osteoblast differentiation and calcium matrix production to promote endochondral osteogenesis (Korstjens et al., 2004). On the other hand, the action of LIPUS on MSCs can increase the expression of type II collagen (Lee et al., 2006), thus promoting the differentiation of mesenchymal stem cells to promote tendon bone healing. A novel finding is that a potential mechanism for LIPUS treatment of tendon-bone interface repair may be macrophage polarization, dependent on phenotypic changes in macrophages (Xu et al., 2022), which may expand the field of clinical trials at a faster rate. Although the LIPUS mechanism has been more intensively studied, it has not yet been confirmed in human studies. Therefore, further clinical trials are recommended to promote early activity and active rehabilitation of patients.

### Mechanical stress

Mechanical loading is an important element in soft tissue healing (Candiani et al., 2010; Khoshgoftar et al., 2011). Some studies have shown that in a goat partial patellectomy mode, the use of tensile load to promote bone-tendon healing was found to promote hyaline cartilage transformation into fibrocartilage, thereby accelerating the healing of the bone-tendon junction and the recovery of the fibrocartilage overlap zone (Wong et al., 2003). One experiment investigated the effect of stress shielding on the patellar tendon in a rabbit patellar tendon model using transverse tension and stress relaxation tests. The results indicated that stress shielding significantly decreased the tangent modulus, tensile strength, and strain at failure of collagen fascicles. Stress shielding significantly alters the transverse tensile and viscoelastic properties of the patellar tendon (Yamamoto et al., 2000). In a rat ACL reconstruction model, higher initial graft tension facilitates recovery of knee laxity and promotes graft healing in ACL reconstruction using free tendon grafts (Fu et al., 2013). It has been shown that mechanical loading can positively affect tendon healing by improving the biomechanical and collagen properties of the Achilles tendon earlier in the healing process, but that this effect diminishes over time (Khayyeri et al., 2020) Figure 2C. It is reasonable to speculate that mechanical loading may have the same effect on tendon bone healing, although this is subject to further verification. However, some studies have found that mechanical loads can also have adverse effects. A rat model of chronic rotator cuff tears was used to investigate the effect of repair tension on tendon-bone healing, showing that repair tension increased with detachment time, stimulating an increase in the amount of poor-quality scar tissue produced and thus adversely affecting tendon-bone healing (Gimbel et al., 2007). In conjunction with current research, the reason for these changes may be that increased repair tension may disrupt the insertion site and prevent proper integration of the tendon with the bone, while stimulating cellular activity and producing poor quality scarring, all of which need to be confirmed by further research (Gimbel et al., 2007). A study of repair tension in a rat ACL reconstruction model found that repair tension adversely affects vascular remodelling in grafts (Carbone et al., 2016). Therefore, patients with rotator cuff tears undergo surgery to repair tension due to minimization. Because the potential mechanism of action of repair tension leading to these changes has not yet been identified, it can be further explored in the future. Histological analyses of numerous animal experiments showed that compressive stress promotes cartilage formation and mechanical tension promotes the process of tendon-bone healing; however, shear force has little effect on the regeneration of the tendon-bone interface (Yamakado et al., 2002; Song et al., 2017). There are relatively few studies on the effect of mechanical strength on tendon-bone healing, and a related study using a patellar tendon repair model showed that low-intensity loads had better healing effects than high-intensity loads (Hettrich et al., 2014). In the future, it is possible to study the effects of mechanical loading in terms of time, amplitude and duration on tendon-bone healing (The specific effects of mechanical stress on tendon-bone healing are listed in the Table 1).

### Other stimulation

According to previous studies, electrical stimulation, combined magnetic fields and exercise therapy have been shown to have a beneficial effect on tendon bone healing. In an experiment on low-frequency electrical stimulation after Achilles tendon resection and suturing in rats, it was observed histologically that collagen 1 and 3 were higher in the group receiving electrical stimulation than in the control group, so low-frequency electrical stimulation could promote collagen expression and increase the amount of collagen (Casagrande et al., 2021) Figure 2D, so it was further suggested that low-frequency electrical stimulation could promote tendon bone healing by promoting collagen expression. It has been shown that electrical stimulation can promote tissue regeneration by promoting cell proliferation and extracellular matrix production, cytokine secretion and vascular development (Ferrigno et al., 2020). However, very high intensity electrical stimulation can lead to cell death (Chen C. et al., 2019). Studies on combined magnetic fields are also relatively rare. When combined magnetic fields were applied to a rabbit patellar partial resection model, the area, length and bone density of newly formed bone were found to be significantly greater than those of the control group (Hu et al., 2015) Figure 2E. The combined magnetic field has been shown to enhance patello-patellar tendon healing by improving endochondral osteogenesis as well as fibrocartilage regeneration and remodelling (Xu et al., 2014). Similarly, postoperative immobilization and early passive motion in a rotator cuff injury model can protect the tendon bone interface and prevent stiffness, so delaying early passive motion is not harmful to tendon bone healing (Zhang et al., 2013). In a recent study, it was shown that early running exercise can delay rotator cuff healing by inactivating Wnt/β-catenin signalling through increased expression of neuropeptide Y (Chen Y. et al., 2021). This shows that the timing of the start of exercise is very important and needs to be further defined. Running exercise has also been shown to significantly increase IL-4 production, activation of the macrophage JAK/STAT signaling pathway, macrophage polarization, and the quality of tendon bone healing (Liu et al., 2022) Figure 2F. Although all of these treatment options are effective to a certain extent, there are some limitations. Because there are relatively few studies on these *in vitro* stimulations, optimising the current treatment remains a challenge in the absence of definitive results. (The specific roles are listed in Table 1).

## Mechanisms of force-regulated tendon-bone healing

### Transcription factors

**FIGURE 3:**
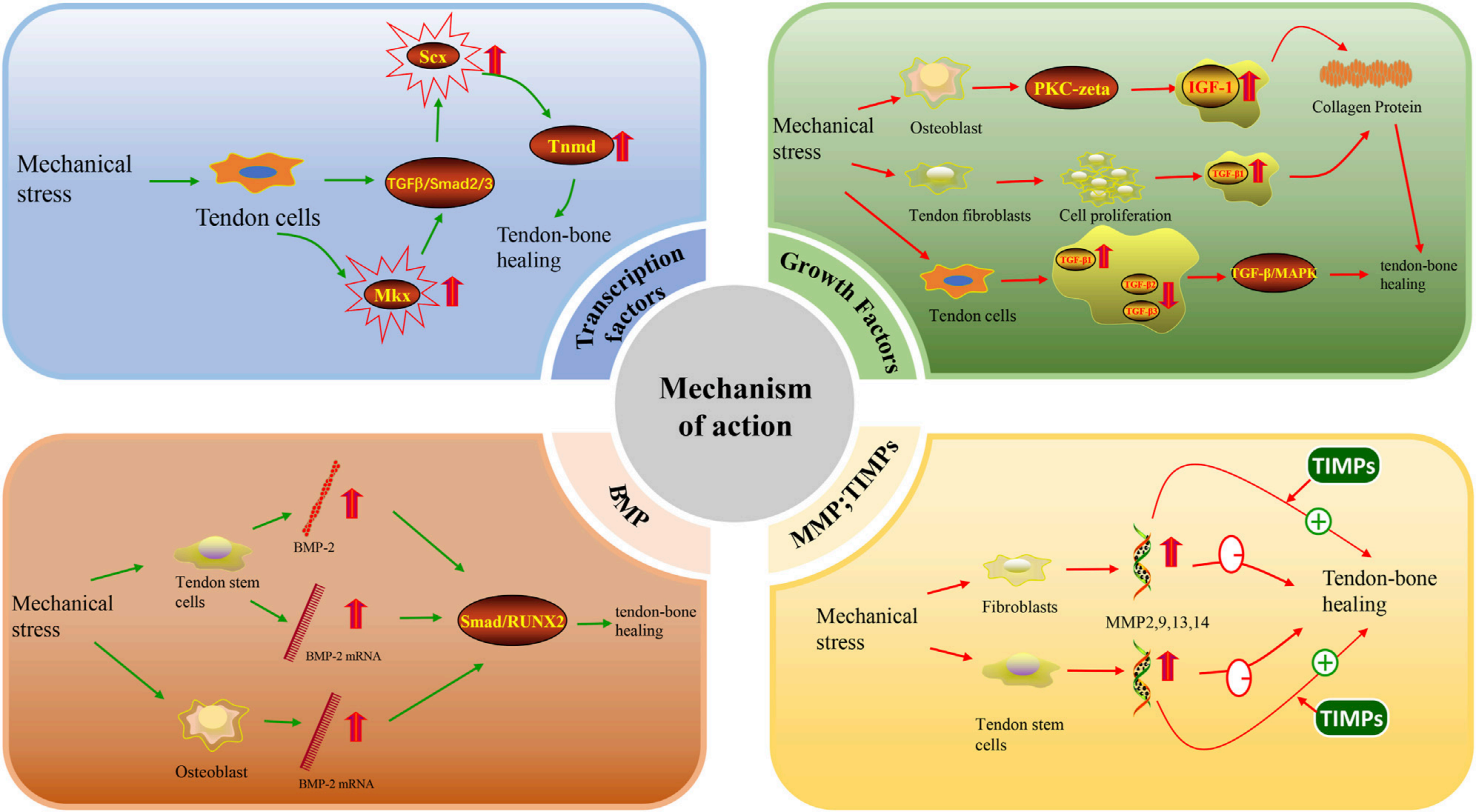
Mechanism of action of force stimulation of tendon bone healing.

Transcription factors are proteins that participate in the initiation or regulation of target gene transcription by binding to the corresponding promoter region of the target gene (Nakamichi and Asahara, 2021). An increasing number of studies show that transcription factors are involved in mechanical stress regulation of tendon-bone, tendon and ligament healing processes. Two major tendon-specific transcription factors, Scleraxis (Scx) and Mohawk (Mkx), have been identified to be regulated by mechanical stress. Scx is the first transcription factor reported to play an important role in tendon differentiation (Cserjesi et al., 1995). Scx is expressed in tendon progenitor cells and remains highly expressed throughout the differentiation of tendon cells into mature tendon cells (Schweitzer et al., 2001). During tendon differentiation and development, Scx regulates Tenomodulin (Tnmd) to some extent (Liu et al., 2021). Previous studies have shown that overexpression of Scx contributes to bone formation (Agarwal et al., 2017). In addition, studies in recent years have found that Scx (+) progenitor cells positively promote the formation of tendon-bone attachment sites (Blitz et al., 2013; Ideo et al., 2020). Scx has also been shown to be necessary for the development of tendon-bone attachment points (Killian and Thomopoulos, 2016). These studies alone suggest that Scx actively participates in and promotes tendon-bone healing. Scx and other tendon-related genes are elevated when mechanical stress is applied to tendon cells (Yang et al., 2019). One study further found that tension can lead to upregulation of Scx expression more so than compression (Takimoto et al., 2015). Mechanical stress can also maintain Scx expression through the TGF-β/Smad2/3 pathway (Maeda et al., 2011). Another transcription factor, Mkx, has been shown to be expressed in tendon and ligament tissues throughout the body of adult mice (Ito et al., 2010). However, the current study suggests that Mkx has both inhibitory (Anderson et al., 2009; Anderson et al., 2012) and activating (Liu et al., 2015; Otabe et al., 2015; Suzuki et al., 2016) effects. Because Mkx, as an inhibitory factor, controls muscle differentiation (Chuang et al., 2014), only the role of Mkx as an activating factor is discussed here. Mkx is overexpressed in human mesenchymal stem cells and can activate the TGF-β signalling pathway to promote elevated Scx expression (Liu et al., 2015; Otabe et al., 2015). The ability of Scx to actively participate in tendon-bone healing has been discussed previously. Mkx can indirectly regulate tendon-bone healing by activating the TGF-β signalling pathway to elevate Scx expression (Suzuki et al., 2016) (Figure 3). In summary, mechanical stress can promote tendon-bone healing by promoting the expression of the transcription factors Scx and Mkx, but their molecular mechanisms have not been elucidated.

### Biological factors

#### Growth factors

IGF-1 and TGF-β both promote tendon-bone healing to some extent. IGF-1 stimulates the proliferation of various cell types, including tendon cells (Tsuzaki et al., 2000). IGF-1 stimulates collagen synthesis in fibroblasts, including tendon cells, and collagen synthesis is dependent on mechanical loading of the tendon (Hansen et al., 2013). Some studies have shown that IGF-1 stimulates collagen production indirectly through TGF-β1 (Ghahary et al., 2000). Topical administration of IGF-1 promotes collagen synthesis in equine tendons and improves healing *in vivo* (Durgam et al., 2012). This shows that IGF-1 promotes collagen synthesis, cell proliferation and protein synthesis and exerts anabolic effects on tendon healing. Regarding the effect of mechanical stress on IGF-1, it has been shown that mechanical stress upregulates the production of IGF-1 mRNA and related proteins in a time-dependent manner (Yan et al., 2021). Both cyclic stretching and intermittent mechanical stress can significantly increase IGF-1 expression (Tang et al., 2006; Pumklin et al., 2015). It has been further shown that fluid shear promotes IGF-1 signalling in osteoblasts in a PKA-zeta-dependent manner (Triplett et al., 2007). In addition, stretching stimulates the autocrine secretion of IGF-1 (Perrone et al., 1995; Xian et al., 2007). In conclusion, multiple modalities of mechanical loading can elevate the expression of IGF-1.

Upregulation of TGF-β expression in the New Zealand White Rabbit model promotes tendon-bone healing after ACL reconstruction by regulating TGF-β/MAPK signalling (Wang et al., 2017). There are many isoforms of TGF-β, among which TGF-β1 promotes cell proliferation and migration as well as type III collagen production to promote tendon-bone healing (Kashiwagi et al., 2004; Kim et al., 2011). In contrast, TGF-β3 expression was associated with reduced scar tissue formation after tendon repair (Kovacevic et al., 2011). In a rat model of rotator cuff repair (Kovacevic et al., 2011), it was reported that early postoperative enhancement with bone-conducting calcium-phosphate matrix at the healing tendon-bone junction led to new bone formation, increased fibrocartilage, and improved collagen organization. The addition of TGF-β3 significantly improved the strength of the repair 4 weeks after the operation and produced a more favourable type I/III collagen ratio. In a study on an *in vitro* fluid shear stress model to mechanically load tendon cells isolated from rats, it was found that fluid shear stress increased TGF-β1 expression but decreased TGF-β2 and TGF-β3 expression (Fong et al., 2005). In addition, the results of cyclic stretching of human tendon fibroblasts under serum-free conditions showed that mechanical stretching in the absence of serum promoted the proliferation of human tendon fibroblasts, upregulated the expression of collagen mRNA and increased the cellular production of type I collagen, part of which was mediated by mechanical stretching to promote the expression of TGF-β1 (Yang et al., 2004). This study also demonstrated that TGF-β1 mRNA expression increased with increasing stretch (Yang et al., 2004). Similar experiments have been performed previously, also by cyclic stretching of human tendon cells, and elevated concentrations of TGF-β were found (Skutek et al., 2001). These results emphasize that tendon mechanical loading induces collagen expression with TGF-β1 and IGF-I as mediators, thereby promoting tendon healing. In summary, mechanical stress can indirectly promote tendon-bone healing by increasing the expression of IGF-1 and TGF-β in various ways (Figure 3). However, studies on this mechanism need to be further validated.

#### Bone morphogenetic protein (BMP)

BMP may be regulated by mechanical loading. It has been shown that uniaxial cyclic tensile strain is sufficient to increase the expression of BMP-2 (Susperregui et al., 2008). In another study, cyclic tensile strain increased BMP-2 gene expression threefold after 6 h of mechanical loading (Yang et al., 2010). In a study exploring the effect of repeated stretching of rat patellar muscle isolated tendon-derived stem cells (TDSCs) on BMP-2 expression, it was shown that BMP-2 expression was increased in the 4% and 8% stretching groups and that BMP-2 mRNA expression was upregulated in the 4% stretching group (Rui et al., 2011). Other studies have shown that the expression of BMP-2, -6, and -7 mRNA is upregulated in osteoblast cell lines subjected to mechanical stretching, but the expression of BMP-4 does not reflect mechanical loading (Siddhivarn et al., 2007). In summary, some subtypes of the BMP family are regulated by mechanical load, but some subtypes are not, such as BMP-4. Therefore, further studies are needed to investigate which members of the BMP family are regulated by mechanical regulation. The effect of BMP on tendon-bone healing has been demonstrated in numerous studies. In a study of a rotator cuff injury model, BMP-2 promoted tendon-bone healing of rotator cuff tears *via* the Smad/RUNX2 pathway (Han et al., 2022) (Figure 3). In another rat ACL reconstruction model, combined treatment with BMP-2 and platelet-rich fibrin (PRF) facilitated increased levels of angiogenesis-promoting growth factors and alleviated the inflammatory response, thereby promoting tendon-bone healing (Han et al., 2019). BMP-4 (Chen H. et al., 2021) and BMP-7 (Yu et al., 2007) have also been shown to have an effect on tendon-bone healing. Therefore, it is reasonable to assume that mechanical loading can upregulate the expression of isoforms such as BMP-2 and BMP-7 and then further promote tendon-bone healing.

#### Matrix metalloproteinases (MMPs) and tissue inhibitors of matrix metalloproteinases (TIMPs)

Previous literature suggests that mechanical loading is capable of regulating MMP expression. For example, in studying the expression of MMP in isolated tendon stem/progenitor cells (TSPCs) from Achilles tendon in response to three different intensities of mechanical stress, it was found that the gene levels of MMP1, MMP2, and MMP3 in mechanically stimulated cells were similar to those in unstimulated cells, indicating that MMP1, MMP2, and MMP3 do not respond to mechanical stress. In contrast, the expression of MMP9, MMP13, and MMP14 was elevated in stretched cells and was independent of the magnitude of the applied stress (Popov et al., 2015). Repeated mechanical loading of fibroblasts revealed that MMP2 expression and activity were upregulated and that high frequency stimulation resulted in greater MMP2 upregulation (Huisman et al., 2016). Other studies and experiments have shown that in tendon cells (Sasaki et al., 2007) and osteoblasts (Wang et al., 2013), mechanical loading leads to elevated expression of MMP.

However, excessive expression of MMPs may disrupt tendon-to-bone healing. MMPs can focus on degrading components of the tissue extracellular matrix and have been shown to play a key role in tissue remodelling (Bedi et al., 2010). In particular, MMP9 and MMP13 contribute to the degradation of the extracellular matrix after injury, where MMP3, MMP4, and MMP14 are involved in matrix interpretation and mechanism remodelling during the healing process (Voleti et al., 2012). Previous studies have shown that the use of TIMPs, which are natural inhibitors of MMPs, may lead to a more mature and organized tendon-bone interface for healing in a rat rotator cuff repair model (Bedi et al., 2010) (Figure 3). It is thus clear that overexpression of MMPs adversely affects tendon-bone healing, but when both MMPs and TIMPs are kept in dynamic balance, effective tendon-bone healing can be promoted. It has been previously described that mechanical loading is able to modulate elevated expression of MMPs, but overexpression of MMPs in turn inhibits tendon-bone healing. Therefore, mechanical loading may in some ways cause MMP overexpression to have a detrimental effect on tendon-bone healing. Therefore, the use of mechanical loading to promote tendon-bone healing could be accompanied by the addition of TIMPs to reduce the overexpression of MMPs. However, this idea needs to be further investigated.

### Common gene expression

Tendon-bone healing is a complex process that involves many genes regulated to some extent by forces. As mentioned previously, the role of genes such as Scx, Mkx, IGF-1, TGF-β, MMP, and BMP in tendon-bone healing has been demonstrated, and all are also affected by mechanical loading. All four groups of BMP genes (OP-1/BMP-7, GDF-5/CDMP-1/BMP-14, GDF-6/CDMP2/BMP-13, and GDF-7/CDMP-3/BMP-12) are expressed during healing, but their modes of expression are different (Eliasson et al., 2008). This shows that gene expression during tendon-bone healing is complex, and some genes can be regulated by forces, but the gene expression patterns may be different during the healing process and need further investigation.

## Conclusion and prospects

In recent years, there has been increasing interest in research on tendon-bone healing after the treatment of rotator cuff injuries and ACL injuries. The aim is for the patient to be able to return to the preinjury level of function and improve the patient’s quality of life. Therefore, research on tendon-bone healing is necessary, especially regarding what modalities are available to promote it. This paper focuses on studies related to the promotion of tendon-bone healing by external stimulation, such as ESWT, LIPUS, and mechanical loading. The focus is on the possible mechanisms of action of force on tendon-bone healing. This study provides stronger evidential support for subsequent mechanical stress of tendon-bone healing. However, since the therapeutic effects of ESWT and LIPUS have not been thoroughly validated in the clinical setting, further strengthening of clinical trials in this area is needed. In contrast, relatively less research has been done on the mechanism of action of force-stimulated tendon-bone healing, but for the transcription factors Scx and Mkx, which promote tendon-bone healing, the expression of these transcription factors is upregulated after stimulation by mechanical stress, as demonstrated by the study that mechanical stress maintains Scx expression through the TGF-β/Smad2/3 pathway (Maeda et al., 2011). The biomolecules IGF-1, TGF-β, and BMP, which promote tendon-bone healing, are also regulated by mechanical loading to some extent, which can upregulate the expression of IGF-1, TGF-β, and BMP mRNA. It is therefore reasonable to assume that force can promote tendon-bone healing by regulating the expression of transcription factors, biomolecules, and genes related to tendon-bone healing. However, more experimental studies in this area are needed to test this hypothesis. Interestingly, it has also been found that force is able to modulate the upregulation of MMP expression, while MMPs may adversely affect tendon-bone healing to some extent, but the adverse effects can be mitigated by the use of TIMPs. It is thus clear that there is likely an adverse effect of force on tendon-bone healing, to be demonstrated by further studies.

It has been shown that force can promote tendon-bone healing. Therefore, force stimulation is a guide to the clinical treatment of tendon-bone healing. There are early studies on ACL reconstruction for tendon-to-bone healing, such as a trial of cyclic loading using four different fixations in ACL reconstruction, which showed that rehabilitation should not be too aggressive during tendon-to-bone healing and that mechanical loading should not be performed immediately after surgery, as this could cause ACL laxity (Giurea et al., 1999). Further studies found that circulatory loading at the time of surgery restored the initial elongation of the graft (Giurea et al., 1999). A subsequent study further confirmed that delayed loading leads to better organization of the tendon-bone interface (Hettrich et al., 2014). Therefore, the force applied to the ACL reconstruction should be minimized during the tendon-to-bone healing process, and it is also important that the timing of mechanical loading be further determined. However, the timing, intensity and duration of force on tendon-bone healing is also very important. Further research is needed to determine the optimal timing, intensity and duration of action to achieve maximum tendon-bone healing at the time of clinical treatment.

Tendon-bone healing is a complex process involving many cells and their survival in the microenvironment. Previous studies on bone marrow stromal cells (Shi et al., 2020), bone marrow-derived stem cells (Chen W. et al., 2021), and bone marrow mesenchymal stem cells (Huang et al., 2020) have shown that these cells are effective in accelerating tendon-bone healing. Recently, a rather surprising finding was the existence of a delicate balance of the internal environment during tendon healing, i.e., a conflict between tendon stem cells and fibro-adipogenic progenitor (FAP) cells. FAP cells are mesenchymal stem cell-like progenitors found in many tissue types and differentiate into lipogenic, chondrogenic and osteogenic cells. Tendon stem cells differentiate into cartilage and osteoblasts but not into adipocytes (Bi et al., 2007). Better tendon-bone healing can only be achieved when the two are in balance. Therefore, future research should capture the balance between these two types of cells. It has been shown previously that mechanical stress has an effect on tendon stem cells (Govoni et al., 2016); however, more research is needed in this area. Similarly, there are even fewer studies on the effect of mechanical stress on FAP cells. Interestingly, studies have provided evidence that platelet-derived growth factor receptor alpha (PDGFRα) signalling promotes tendon regeneration and fibrosis. The authors conditionally knocked down PDGFRα in tubulin polymerization-promoting protein family member 3-expressing (Tppp3+) cells and tracked the fate of the cells to test whether PDGFRα signalling was required for tendon regeneration. The results confirmed that PDGFRα signalling promotes the proliferative capacity of tendon stem cells and is important for tendon cell differentiation (Harvey et al., 2019). It has been shown that when pressure is applied to the periodontal ligament, the PDGFRα signal gradually increases and then decreases (Zhang et al., 2016). However, it is still unknown whether mechanical stress can modulate PDGFRα signalling during tendon-bone healing. Future studies can focus on these two aspects.

In conclusion, accelerating tendon-bone healing has been shown to be possible with a variety of *in vitro* stimulation. *In vitro* stimulation is non-invasive, simple to perform and effective and therefore has a high appeal in promoting tendon-bone healing. However, these findings have been made in limited experimental studies. In future trials, there are still many issues to be resolved before clinical use, such as application technique, duration and timing of action. Also, the mechanism of action of *in vitro* stimulation on tendon-bone healing and whether mechanical stress modulates PDGFRα signalling will have to be investigated.
